# Gonadotropin-releasing hormone agonist triggering of oocyte maturation in assisted reproductive technology cycles

**DOI:** 10.4274/tjod.92979

**Published:** 2015-06-15

**Authors:** Engin Türkgeldi, Lale Türkgeldi, Ayşe Seyhan, Barış Ata

**Affiliations:** 1 Koç University Hospital, Department of Obstetrics and Gynecology, İstanbul, Turkey; 2 Kanuni Sultan Süleyman Research and Teaching Hospital, Clinic of Obstetrics and Gynecology, İstanbul, Turkey; 3 American Hospital, Assisted Reproduction Unit, İstanbul, Turkey; 4 Koç University Faculty of Medicine, Department of Obstetrics and Gynecology, İstanbul, Turkey

**Keywords:** Ovarian hyperstimulation syndrome, hCG, GnRH agonist, in vitro fertilization, luteal phase

## Abstract

Gonadotropin-releasing hormone agonists (GnRHa) have gained increasing attention in the last decade as an alternative trigger for oocyte maturation in patients at high risk for ovarian hyperstimulation syndrome (OHSS). They provide a short luteinizing hormone (LH) peak that limits the production of vascular endothelial growth factor, which is the key mediator leading to increased vascular permeability, the hallmark of OHSS. Initial studies showed similar oocyte yield and embryo quality compared with conventional human chorionic gonadotropin (hCG) triggering; however, lower pregnancy rates and higher miscarriage rates were alarming in GnRHa triggered groups. Therefore, two approaches have been implemented to rescue the luteal phase in fresh transfers. Intensive luteal phase support (iLPS) involves administiration of high doses of progesterone and estrogen and active patient monitoring. iLPS has been shown to provide satisfactory fertilization and clinical pregnancy rates, and to be especially useful in patients with high endogenous LH levels, such as in polycystic ovary syndrome. The other method for luteal phase rescue is low-dose hCG administiration 35 hours after GnRHa trigger. Likewise, this method results in statistically similar ongoing pregnancy rates (although slightly lower than) to those of hCG triggered cycles. GnRHa triggering decreased OHSS rates dramatically, however, none of the rescue methods prevent OHSS totally. Cases were reported even in patients who underwent cryopreservation and did not receive hCG. GnRH triggering induces a follicle stimulating hormone (FSH) surge, similar to natural cycles. Its possible benefits have been investigated and dual triggering, GnRHa trigger accompanied by a simultaneous low-dose hCG injection, has produced promising results that urge further exploration. Last of all, GnRHa triggering is useful in fertility preservation cycles in patients with hormone sensitive tumors. In conclusion, GnRHa triggering accompanied by appropriate luteal phase rescue protocols is a relatively safe option for patients at high risk for OHSS.

## INTRODUCTION

Gonadotropin-releasing hormone agonists (GnRHa) have gained increasing attention in the last decade as an alternative trigger for oocyte maturation in patients at high risk for ovarian hyperstimulation syndrome (OHSS). However, their history goes further back. In this paper, we aimed to review the history and current practice of GnRHa triggering in assisted reproductive technology cycles, as well as to explore possible future applications.

In 1973, two years after the discovery of the amino acid sequence of GnRH by Schally and Guillemin, Nakano et al. successfully induced ovulation in humans using synthetic GnRH infusion^(1)^. GnRH agonists were developed in the following years; however, the first studies that examined their use in ovulation induction were performed more than a decade later. The first randomized controlled trial (RCT) on the subject was performed by Gonen and Casper in 1990. In their pilot study, 18 women underwent ovarian stimulation without pituitary suppression to receive GnRHa or human chorionic gonadotropin (hCG) as the trigger to induce final follicular maturation. They reported similar numbers of oocytes collected, fertilization rate, embryo quality, and pregnancy rates^(2)^. Casper reported similar results in a similar study with 179 women^(3)^. However, pituitary suppression with GnRHa to prevent spontaneous ovulation became the norm in the coming years and GnRHa as a trigger remained in oblivion until the introduction of gonadotropin releasing hormone antagonists (GnRHant) into IVF practice.

In 1990, Imoedemhe et al. recognized the potential of GnRHa trigger to prevent OHSS^(4)^. In their study of 38 women who underwent in vitro fertilization (IVF) with peak serum estradiol >4000 pg/mL, they used GnRHa trigger and reported no cases of OHSS. The rationale behind this and many similar studies to follow was that GnRHa use resulted in a milder luteotrophic stimulus than hCG triggering. HCG has been the agent of choice for triggering ovulation in ART cycles. Owing to its common subunit with luteinizing hormone (LH), hCG mimics the LH surge in the natural cycle. It has a significantly longer half-life than LH, and can be detected in the serum up to 10 days after administration, resulting in extended luteotrophic stimulus^(5)^. Although this provides adequate luteal phase support and promotes implantation, it puts the patient at increased risk for OHSS.

To the contrary, GnRHa triggering results in a considerably short-lasting LH surge. LH peaks around 4 hours after administration and rapidly returns to its baseline value in about 20 hours. This is in sharp contrast with the natural cycle, in which it takes LH 14 hours to reach the peak, followed by a 14-hour-long plateau before returning to baseline after 48 hours^(6,7)^. Combined with the short half-life of LH, the short LH peak decreases the luteinizing stimulus on the granulosa cells, limiting the production of vascular endothelial growth factor (VEGF), which is the key mediator that leads to increased vascular permeability, the hallmark of OHSS^(8,11,10)^. However, limited luteinization was found to be associated with significantly decreased estradiol and progesterone production during the luteal phase^(5)^. GnRHa trigger was associated with decreased size and number of corpora lutea, decreased expression of steroidogenic enzymes, as well as decreased expression of molecules that play a role in formation and stabilization of new vessels, such as angiopoietin^(11)^. A recent study in humans confirmed similar findings. It is noteworthy that GnRHa trigger was associated with increased LH receptor expression in cumulus cells^(12)^.

Initial RCTs compared clinical outcomes of GnRHa triggered and hCG triggered cycles in normoresponder patients undergoing IVF^(5,13,14)^. In all three studies, both groups received conventional luteal phase support with progesterone with or without estradiol. These studies confirmed Casper’s findings of similar oocyte yield and embryo quality with both triggers. However, significantly lower pregnancy rates along with significantly higher rates of pregnancy loss were seen in GnRHa triggered groups. The results were so discouraging that Kolibianakis and Humaidan had to terminate their studies prematurely^(13,14)^. Griesinger’s analysis of these 3 studies showed a 7.9% and 29.9% pregnancy rate per randomized patient; and 67.6% and 12.7% rate of pregnancy loss in GnRHa and hCG trigger groups, respectively^(15)^. These worrisome rates were hypothesized to be due to either luteal phase deficiency or poor egg/embryo quality. In a randomized trial of 60 donors and 89 recipients, Acevedo et al. demonstrated similar fertilization, implantation and pregnancy rates in oocyte recipients from donors triggered with hCG or GnRHa^(16)^. Similar retrospective and prospective studies supported these findings^(17,18,19)^. Griesinger electively vitrified pronucleate (2PN) oocytes of 40 patients at high risk for OHSS, and transferred embryos later in an artificial cycle, resulting in comparable live birth rates^(20)^. Altogether, these data provided convincing evidence for a severely defective luteal phase that hampers implantation and delivery rates following GnRHa triggering.

Two methods have been proposed to rescue the luteal phase in GnRHa trigger cycles. The first is administering intensive luteal phase support (iLPS) with high doses of progesterone and estrogen. This approach yielded contradictory results. In their retrospective cohort study, Engmann et al. administered 50 mg/day intramuscular progesterone and 0.3 mg transdermal estrogen every other day starting the day following oocyte pick-up (OPU) to 23 patients with polycystic ovary syndrome (PCOS), polycystic ovaries (PCO) or who were otherwise at high risk of developing OHSS. They monitored estrogen and progesterone levels weekly until the 10^th^ gestational week and increased the progesterone dosage to 75 mg/day if serum progesterone levels dropped below 20 pg/mL. Likewise, if serum estrogen levels fell below 200 pg/mL, they increased dosage to 0.4 mg/day and, if necessary, added oral estrogen to maintain the desired serum level. This active and aggressive support resulted in 65.2% live birth rate, which was comparable to the live birth rate of a similar cohort of women who were given an hCG trigger. There were no cases of OHSS in the study group^(21)^. On the other hand, Babayoff et al. conducted a study with a similar protocol in PCO patients, but had to discontinue the RCT due to unacceptably high rate of early pregnancy loss in the GnRHa trigger arm^(22)^. The time of iLPS initiation may have been an important difference between these studies. Engmann began iLPS the day after oocyte pick-up, whereas Babayof withheld support in the first 48 hours, and this difference may explain the contradictory results. Shapiro et al. reported that early-start iLPS was associated with better outcomes following a GnRHa trigger^(23)^.

Engmann tested his protocol in an RCT that included 66 patients from a similar population, and achieved 53% and 48% ongoing pregnancy rates in GnRHa and hCG trigger cycles, respectively. While there were no cases of OHSS in the GnRHa group, 10 of the 32 patients in the hCG group had OHSS^(24)^. It was suggested that Engmann’s cohort of patients with PCOS had the advantage of higher endogenous LH levels than the normal population and this could have helped rescuing the corpora lutea despite the absence of hCG. Later studies demonstrated that high endogenous levels contribute to iLPS success^(25)^. A retrospective study of 316 GnRHa triggered cycles showed that every one unit increase in LH on the day of trigger was associated with 13% increase in the odds of a clinical pregnancy^(26)^. In a later retrospective study, the same researchers reported that they could not retrieve any oocytes from 19% of patients with serum LH levels below 15 IU/L^(27)^. Chen et al. stratified 91 patients into 6 groups according to their serum LH levels 12 hours after GnRHa triggering. They reported a significantly lower oocyte yield in patients with LH lower than 15.0 IU/L, however, fertilization rates or clinical outcomes were not different for any group^(25)^. Overall, GnRHa triggering followed by immediate iLPS is a reasonable option for women with PCOS, who actually constitute the highest risk group for OHSS following hCG injection, even after small doses.

**Figure 1:**
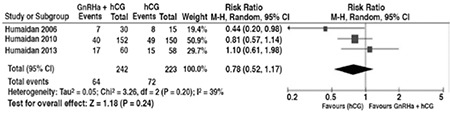
Comparison of ongoing pregnancy rates in conventional hCG trigger and GnRHa trigger+low-dose hCG injection groups in the three RCTs by Humaidan et al. GnRH: Gonadotropin-releasing hormone agonist, hCG: Human chorionic gonadotropin

The other option to overcome luteal phase deficiency following GnRHa trigger is administering low dose adjuvant hCG. In their pilot study, Humaidan et al. found that cycles triggered with 0.5 mg buserelin and supplemented with 1500 IU hCG 35 hours later, along with daily 90 mg vaginal progesterone and 4 mg oral estrogen, provided clinical pregnancy rates comparable with 10.000 IU hCG triggered cycles^(28)^. This outcome encouraged the same team to undertake a large RCT of 302 patients, in which they compared the same protocols that were in their pilot study. The live birth rates were not significantly different, at 24% and 31% for GnRHa and hCG trigger groups, respectively^(29)^. When the data from the three RCTs by Humaidan are combined in a forest plot, ongoing pregnancy rates are not found to be significantly different between conventional hCG trigger and GnRHa trigger with low-dose hCG injection groups, yet hCG trigger is associated with more favorable outcomes^(28,29,30)^ (Figure 1). No cases of OHSS were reported in the GnRHa trigger groups in the first two trials; however, it is noteworthy that both of these studies included normoresponder patients. In two uncontrolled series of high-risk women with 12 and 71 patients, low-dose adjuvant hCG was used. No early onset OHSS was reported in the studies, and 1 late case of OHSS occurred in each, while maintaining satisfactory ongoing pregnancy rates^(31,32)^. Although these studies claimed to totally eliminate early OHSS with GnRHa trigger and low-dose hCG support; however, reports of severe early OHSS with the same protocol have been published. Seyhan et al. reported 6 cases of severe OHSS after administiration of 1500 IU hCG following a GnRHa trigger in their series of 23 high-risk patients^(33)^. This was despite employing additional preventive strategies including coasting, metformin, and cabergoline administration. Moreover, even withholding low-dose hCG and implementing a freeze-all strategy does not guarantee evading early-onset severe OHSS, as 5 such cases were recently reported^(34,35)^. GnRHa triggering with or without low-dose adjuvant hCG or a freeze-all strategy provided great advances in OHSS prevention, but they unfortunately cannot totally eliminate early-onset OHSS.

Research has been lacking for identifying thresholds for ovarian response such as serum hormone levels, or follicle numbers that can guide the clinician in choosing the most appropriate approach for a high-risk patient. In a review paper, Engmann et al. proposed iLPS to be used for patients with estrogen levels greater than 4000 pg/mL, whereas for those with lower estrogen levels, the authors suggested a dual trigger with GnRHa and 1000 IU hCG, followed by iLPS^(36)^. RCTs are required to investigate the validity of this proposal. For the time being, a GnRHa trigger followed by iLPS can be recommended for patients with PCOS, given the high endogenous LH levels and risk of OHSS in these patients, even with low-dose hCG administration. Luteal phase rescue with low dose hCG is a more common and relatively more practical method, despite its low but existent risk of OHSS. Still, the safest approach for women with exaggerated ovarian response appears to be avoiding hCG altogether and cryopreserving embryos for use in a subsequent cycle^(37)^.

Other features of GnRH agonists than preventing OHSS have also been investigated. Castillo et al. conducted a large study examining the incidence of empty follice syndrome (EFS) after a GnRHa trigger^(38)^. The EFS rates of 2034 donors who used GnRHa and 1433 patients undergoing IVF who used 250 microgram recombinant hCG as a trigger were compared and both groups had mildly high but similar EFS rates, 3.5% vs 3.1%, respectively. Later, Kummer et al. reported the EFS rate to be 1.4% in their retrospective study. An interesting observation was that all cases of EFS had low LH (<15 IU/L) and low progesterone (≤3.5 ng/mL) levels following trigger^(27)^. This finding and its significance should be validated with prospective studies.

Budinetz et al. compared congenital anomaly, obstetric and neonatal complication rates in 152 GnRHa and 240 hCG triggered cycles, retrospectively, and reported similar rates for all three outcome measures, which supported the safety of GnRHa triggering^(39)^. However, two small studies of the same researchers reported increased rates of small for gestational age babies and pre-eclampsia in women with high estrogen levels during triggering, which favors a freeze-all strategy for better obstetric and neonatal outcomes^(40,41)^. A recent retrospective study of 466 cycles by Sahin et al. showed increased ectopic pregnancy rates in GnRHa triggered cycles compared with hCG triggered cycles. The authors speculated that this effect could be due to suboptimal endometrial receptivity^(42)^. More data is needed to corroborate this interesting finding.

**Figure 2:**
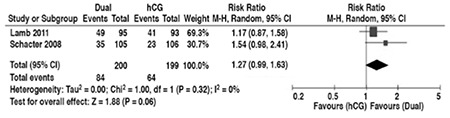
Comparison of ongoing pregnancy or live birth rates with and without follicle stimulating hormone surge. GnRH: Gonadotropin-releasing hormone agonist, hCG: Human chorionic gonadotropin, Dual: Dual trigger

The FSH surge induced by GnRHa trigger matches the natural cycle, which intrigues researchers about its possible function. It has been shown to increase LH receptor expression on granulosa cells^(43)^, which in turn is claimed to improve nuclear maturation and enhance oocyte competence, possibly resulting in improved clinical outcomes^(12)^. However, increased expression of LH receptors may not necessarily be beneficial, as Maman et al. demonstrated in their study that overexpressed LH receptors may interfere with signaling and hinder fertilization^(44)^. Nevertheless, some researchers wondered if they could make use of the potential benefit of the FSH surge while not compromising the luteal phase, and proposed the dual-trigger protocol. In this protocol, a GnRHa trigger is accompanied by a simultaneous low- dose hCG injection. Initially, Shapiro et al. reported a favorable outcome of 53.3% clinical pregnancy rate for dual trigger in their retrospective study of 45 patients^(45)^. Later, Griffin et al. compared the dual-trigger and GnRHa trigger cycles retrospectively in 102 women with estrogen levels below 4000 pg/mL but still at high risk for OHSS. Although number of retrieved oocytes and fertilization rates were similar in both groups, clinical pregnancy and live birth rates were significantly higher in the dual-trigger arm, 58.8% vs 36.8%, and 52.9% vs 30.9%, respectively. The authors commented that this difference may be attributed to the positive effects of the FSH surge^(46)^. The results of the RCT undertaken by Schacter et al. were in line with Griffin’s study, which reported significantly higher ongoing clinical pregnancy rate for the dual-trigger group than the hCG trigger group^(47)^. In a well-designed RCT with 188 patients, Lamb et al. investigated the effect of FSH surge in oocyte maturation. After suppressing endogenous LH and FSH secretion using a long-agonist protocol, they randomized patients either to the treatment group, which received 450 IU FSH simultaneously with 10.000 IU HCG for triggering, or to the placebo group, which received 10.000 IU HCG and placebo. Oocyte yield and fertilization rates were significantly higher in the treatment group (69.9% vs. 57.1% and 0.62 vs 0.48, respectively). However, although there was a trend for higher clinical pregnancy rates in the treatment group (56.8% vs 46.2%), this was not found to be statistically significant ^(48)^. The unified data from the two RCTs that study the effect of FSH surge show that ongoing pregnancy or live birth rates are not, but almost, significantly higher when there is an FSH surge (Figure 2)^(47,48)^. These limited number of studies show that the FSH surge may actually be beneficial, and dual trigger seems to be an option worth exploring.

Another setting where GnRHa triggering could prove useful is fertility preservation cycles in patients with hormone sensitive tumors, such as breast cancer. Rapid luteolysis and lower estradiol and progesterone levels following the GnRHa trigger are hypothesized to provide a safe option for women undergoing IVF for fertility preservation. In Oktay et al.’s retrospective study of women with breast cancer, a GnRHa trigger arm yielded significantly more mature oocytes and higher fertilization rates than the hCG trigger arm and resulted in lower estrogen levels and OHSS rates^(49)^. These findings have been corroborated in a more recent and larger retrospective series by the same authors^(50)^.

In conclusion, GnRHa triggering, when used in conjunction with an appropriate luteal phase rescue protocol, yields similar number of mature oocytes and fertilization rates compared with conventional hCG triggering. Clinical pregnancy rates are comparable, but still tend to be higher with hCG trigger. GnRH agonists should be the trigger of choice for patients at high risk for OHSS; however, the rescue method or the decision to proceed with fresh or frozen transfer should be individualized. More studies are needed to determine thresholds of ovarian response, which would determine whether any LH activity, in the form of hCG or otherwise, should be added and a fresh transfer performed. Until then, luteal phase can be rescued using iLPS in patients with PCOS. Low-dose hCG injection for rescue is a popular and useful method that has reduced OHSS greatly; however, it should be remembered that no method, including the freeze-all strategy, totally prevents OHSS. The safest option to prevent late OHSS is cryopreservation of the embryos. Initial studies with dual trigger reported promising results; however, well-designed RCTs are urgently needed to support those findings.
